# DESIGNING FOR PROGRAM SUSTAINABILITY: AN EVALUATION OF BC’S PROVINCIAL BETTER AT HOME PROGRAM

**DOI:** 10.1093/geroni/igad104.2088

**Published:** 2023-12-21

**Authors:** Camille Hannah, Bobbi Symes, Kahir Lalji

**Affiliations:** United Way British Columbia, Burnaby, British Columbia, Canada; United Way British Columbia, Burnaby, British Columbia, Canada; United Way British Columbia, Burnaby, British Columbia, Canada

## Abstract

Better at Home programs help older adults in communities across British Columbia with non-medical tasks such as light housekeeping, yard work, transportation to appointments, grocery and meal delivery, and friendly visiting, helping them to remain at home and stay connected to their communities. Better at Home is funded by the Province of British Columbia and managed by the United Way. An evaluation of 81 Better at Home programs took place over 2022/23 focusing on the delivery, design, and sustainability of programs. A multipronged evaluation approach was utilized, including site visits; document and administrative data review; interviews with program staff, older adults, and caregivers (n=87); and a survey of program stakeholders (n=399). Over 2021/22, a total of 244,875 services were provided through Better at Home. The most common services provided were light housekeeping, friendly visiting, prepared meal delivery, and grocery services. The evaluation identified design and delivery best practices (e.g., building trust and relationships), program opportunities (e.g., leveraging communities of practice), and lessons learned (e.g., increasing awareness of burnout, capacity, and duplication). Some challenges to program efficiency were also identified (e.g., increased demand for services and complexity of client needs, staff and volunteer turnover). Six program improvements were recommended to ensure the long-term sustainability of Better at Home programs: 1) Securing multi-year funding that accounts for inflation, 2) Ensuring sufficient human resources for service delivery, 3) Offering strategic training opportunities, 4) Continuing to invest in existing programs, 5) Growing partnerships and community networks, and 6) Providing comprehensive supports to program staff.

